# Poliovirus detections in Europe – urgent action needed to keep Europe polio-free

**DOI:** 10.2807/1560-7917.ES.2025.30.4.2500076

**Published:** 2025-01-30

**Authors:** Pamela Rendi-Wagner, Hans Kluge

**Affiliations:** 1 European Centre for Disease Prevention and Control (ECDC), Stockholm, Sweden; 2 World Health Organization Regional Office for Europe (WHO/Europe), Copenhagen, Denmark

**Keywords:** poliovirus, Europe

Polio has threatened the health and wellbeing of children for centuries, but today it is largely a forgotten disease for the vast majority of people living in Europe. Polio vaccination has undeniably been one of the most successful public health interventions in Europe and worldwide. Thanks to successful immunisation programmes, surveillance and outbreak response, Europe was declared free of endemic polio in 2002 and has since maintained this status every year [1]. 

Nonetheless, until global eradication is achieved and as long as poliovirus is circulating anywhere, importations into Europe are inevitable. Data from poliovirus surveillance systems show that pathogenic poliovirus was detected in at least one of the countries in Europe in every year from 2015 through 2022 [2].

It is paramount that we see every importation of any harmful poliovirus into Europe as a wake-up call – polio may be forgotten by many, but it is not gone, and it still poses a risk to the unvaccinated in Europe and every other region in the world.

The unusually high number of poliovirus detections in recent months has reinforced the urgency of the wake-up call. From September through December 2024, vaccine-derived poliovirus type 2 (VDPV2) was detected in wastewater systems of 14 cities in five European countries [3-5]: consecutively in Spain, Poland [6], Germany, the United Kingdom (UK), and Finland. The isolated VDPV2 viruses are linked to a lineage (NIE-ZAS-1) that was first detected in Nigeria in July 2020, and which has travelled in the intervening years to 21 other countries in Africa, causing outbreaks in 15 of them (WHO data, not shown) [7].

Genetic sequencing analysis of the European isolates identified divergence from the closest known NIE-ZAS-1 isolates, indicating that before detection in Europe, the virus had been circulating elsewhere undetected for ca 1 year. Additionally, and remarkably, the analysis indicated wide genetic variation between the European isolates, both between countries, and within countries, and even between isolates from the same sampling site [3,4]. This suggests an unusual event in which multiple nearly simultaneous and apparently independent importations likely occurred, from a location outside the catchment areas of European poliovirus surveillance networks.

To date, there is no evidence that widespread local circulation has occurred in Europe from the 2024 VDPV2 importations described above; however, importations of polioviruses can and do lead to outbreaks if the virus finds its way to unvaccinated individuals.

This recently occurred in Tajikistan [8] and Ukraine [9] in 2021, and Israel [10] and the UK [11] in 2022. In 2022 and 2023, VDPV2 outbreaks affected countries which had high overall vaccination coverage but localised pockets of under-immunised communities with tight social linkage; in addition to Israel and the UK, genetically linked VDPV2 isolates were detected in Canada and the United States, and the outbreak resulted in two paralytic cases of polio in unvaccinated individuals in New York State [12] and North Israel [13].

Those outbreaks were a painful reminder that despite overall high national coverage, as long as countries have sub-populations that are under-vaccinated, the steady stream of poliovirus importations could find fertile ground leading to circulation and to paralytic cases. This can and must be prevented.

In general, the five countries reporting VDPV2 detection in 2024 have maintained high national immunisation coverage with three doses of inactivated poliovirus vaccine (IPV), but all countries have experienced disparities in sub-national communities (in Germany, Finland, Poland, Spain and the UK collectively, sub-national coverage of the third dose of polio-containing vaccine in 2023 ranged from 43% to > 99%), leaving some populations chronically under-vaccinated and dangerously vulnerable to poliovirus infection and risk of paralysis if exposed to imported virus. Within the European Union and European Economic Area (EU/EEA), the European Centre for Disease Prevention and Control (ECDC) estimated that during the 10-year period from 2012 to 2021, ca 2.4 million children between 12 and 23 months of age may not have received the required three doses of polio-containing vaccine to ensure they are protected [14]. Updated estimates have added another 600,000 children for the years 2022 and 2023 who may have missed their vaccination [15]. To minimise the risk of outbreaks, countries have to take urgent action to identify un- and under-vaccinated individuals and sub-populations and develop intensified and innovative programmes to fill these immunity gaps.

The ECDC most recently published a Rapid Risk Assessment on these multiple detections that sets out the key priority actions that all countries in Europe should undertake, urgently, to prevent or curb any possible local transmission of polioviruses [15]. The World Health Organization (WHO) Regional Office for Europe has also published guidance to support countries in identifying, addressing and tracking inequities in immunisation, following a step-wise approach that includes data triangulation and analysis, formative research to identify barriers to vaccination, and tailored programme policies, strategies and practices to increase uptake [16].

A future without polio remains our goal, but it is by no means a certainty. The continuing spread of polioviruses globally and the increased frequency of poliovirus detections in the past year in Europe and neighbouring regions make the path towards eradication look increasingly fragile. As laid out in the European Immunization Agenda 2030 [17], the GPEI Polio eradication strategy [18] and the Global Polio Surveillance Action Plan 2025–2026 [19]: **every country** must remain **vigilant** - to detect the presence of polioviruses through sensitive surveillance systems; **prepared** - to act quickly if any circulation is detected; and **committed** - to sustain high vaccination coverage in every community every year until global polio eradication has been achieved. Lack of sustained progress in any of these areas heightens the risk of a polio outbreak, and with it the potential for sustained transmission, loss of our polio-free status and a major setback on the path towards global eradication.

The WHO and ECDC continue their close cooperation to support national and local public health authorities in these efforts, including the provision of technical guidance and resources for effective surveillance and outbreak response, facilitating data exchange and genetic sequencing for tracking of poliovirus strains, and assisting in the development and implementation of targeted risk communication and community engagement strategies to increase vaccine acceptance and enhance immunisation uptake, all while maintaining vigilant and ongoing monitoring of the situation [20-22].

The last stages of the global polio eradication strategy grapple with the outstanding challenges of eliminating wild polioviruses in endemic countries on the one hand and putting a halt to outbreaks caused by circulating vaccine-derived polioviruses (cVDPVs) on the other. In addition, we must do all we can to prevent, by all means, the resurgence of polio in polio-free areas. Europe remains committed to do its part in the context of all relentless global efforts in this direction and has full capacity to do so successfully.

## Note

‘Europe’ is used in this editorial to refer to the 53 Member States of the WHO European Region, which includes the 27 member countries of the European Union.

Pamela Rendi-Wagner is the Director of the European Centre for Disease Prevention and Control (ECDC) and Hans Kluge is the World Health Organization (WHO) Regional Director for Europe.
